# The Significance of Genetically Determined Methylation and Folate Metabolism Disorders in the Pathogenesis of Coronary Artery Disease: A Target for New Therapies?

**DOI:** 10.3390/ijms25136924

**Published:** 2024-06-25

**Authors:** Agnieszka Pietruszyńska-Reszetarska, Robert Pietruszyński, Robert Irzmański

**Affiliations:** 1 Department of Internal Medicine, Rehabilitation and Physical Medicine, Medical University of Lodz, 90-645 Lodz, Poland; robert.irzmanski@umed.lodz.pl; 2 Cardiology Outpatient Clinic, Military Medical Academy Memorial Teaching Hospital of the Medical University of Lodz—Central Veterans’ Hospital, 90-549 Lodz, Poland; robpiet-portal@wp.pl

**Keywords:** methylation, folate, 5-MTHF, coronary artery disease, biomarker, personalized medicine

## Abstract

Methylation is a biochemical process involving the addition of a methyl group (-CH_3_) to various chemical compounds. It plays a crucial role in maintaining the homeostasis of the endothelium, which lines the interior surface of blood vessels, and has been linked, among other conditions, to coronary artery disease (CAD). Despite significant progress in CAD diagnosis and treatment, intensive research continues into genotypic and phenotypic CAD biomarkers. This review explores the significance of the methylation pathway and folate metabolism in CAD pathogenesis, with a focus on endothelial dysfunction resulting from deficiency in the active form of folate (5-MTHF). We discuss emerging areas of research into CAD biomarkers and factors influencing the methylation process. By highlighting genetically determined methylation disorders, particularly the *MTHFR* polymorphism, we propose the potential use of the active form of folate (5-MTHF) as a novel CAD biomarker and personalized pharmaceutical for selected patient groups. Our aim is to improve the identification of individuals at high risk of CAD and enhance their prognosis.

## 1. Introduction

In cardiovascular diseases (CVD), significant progress has been made in novel diagnostic and treatment methods. These include interventions in cardiology and the management of post-hospitalization patients through hybrid, comprehensive cardiac rehabilitation using telemedicine. This approach has been shown to enhance the quality of life for patients with heart failure (The Telerehabilitation in Heart Failure Patients Trial, TELE-REH-HF) [1].

The primary objective of innovation in cardiology is to reduce the incidence of CVD and Major Adverse Cardiovascular Events (MACE), often defined as myocardial infarction, cardiac arrest, coronary revascularization procedure, ischemic stroke, or death from cardiovascular causes. Despite extensive research, including multi-omics data (including genomics, epigenomics, transcriptomics, proteomics, and metabolomics) along with artificial intelligence (AI), CVDs are widespread in the general population [2].

The identification of prognostic biomarkers in primary CAD prevention and the management of MACE in CAD patients during secondary prevention are crucial for enhancing patient management and outcomes. Presently, CAD prevention strategies primarily target traditional risk factors such as obesity, diabetes, hypertension, and dyslipidemia. However, there exists a notable gap in addressing additional risks posed by non-traditional factors [3].

So far, the majority of the genetically inspired drugs for CAD patients have been associated with lipid metabolism. Although the clinical results are promising, they do not represent all new pathophysiological concepts.

The impaired methylation pathway, being a complex network of connections between the folates (such as folate and 5-methyltetrahydrofolate), one-carbon tetrahydrobipterin cycles, and the transsulfuration pathway, has been gaining interest with regard to CAD caused by genetic polymorphism of the methyltetrahydrofolate reductase gene (*MTHFR*). However, none of the biomarkers of this process (e.g., homocysteine) have been widely used in clinical practice. Instead of other biomarkers involved in the methylation process, the 5-methyltetrahydrofolate (5-MTHF) concentration in the blood is a reflection of the physiological form of folate in the blood, which has already undergone intestinal absorption and enzymatic conversion.

The field of pharmacogenomics, which focuses on individual drug selection based on the genetic polymorphisms and mutations of each patient, is rapidly advancing. Patients with genetic methylation disorders may be suitable candidates for personalized therapy utilizing the active form of folate.

The aim of this review is to clarify the physiological importance of the methylation pathway and folate metabolism in CAD patients, as this area is not yet widely understood. We aim to explore how this knowledge could be utilized to uncover novel, personalized treatment approaches for patients.

## 2. Overview of the Methylation Pathway

Methylation is a biochemical process in which a methyl group (-CH_3_) is attached to chemical compounds such as neurotransmitters, lipids, proteins, and DNA [4].

**Figure 1:**
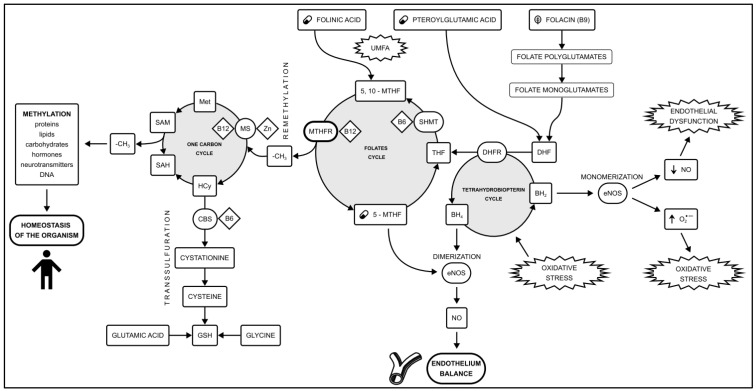
The methylation pathway is formed by the one-carbon cycle and the folate cycle. An additional role is played by the transsulfuration pathway and the tetrahydrobiopterin cycle. Methylation disorders caused by the *MTHFR* polymorphism play a role in the development of vascular endothelial dysfunction. 5-MTHF availability plays a key role in the amount of circulating NO. Shifting the balance between NO production and oxidative stress in endothelial cells is a key step in CAD development. Based on [23,24,25]. Abbreviations used: (O_2_^•−^) Superoxide anion radical; 5,10-MTHF—5,10-methylenetetrahydrofolate; 5-MTHF—methylated folate, levomefolic acid; BH_2_—Dihydrobiopterin; BH_4_—Tetrahydrobiopterin; CAD—coronary artery disease; CBS—cystathionine β-synthase; DHF—dihydrofolate; DHFR—dihydrofolate reductase; eNOS—endothelial nitric oxide synthase; GSH—reduced glutathione; Hcy—homocysteine; Met—methionine; MS—methionine synthase; *MTHFR*—methyltetrahydrofolate reductase; NO—nitric oxide; SAH—S-adenosylhomocysteine; SAM—Sdenosylmethionine; SHMT—serine hydroxymethyltransferase; THF—tetrahydrofolate; UMFA—unmetabolized folic acid syndrome.

The main metabolic pathways involving methylation are the folate cycle and the one-carbon group cycle [5]. These pathways are essential for generating methyl groups to regulate all methylation-related processes associated with metabolic processes governing cell division and tissue development. An additional role in this pathway is played by the transsulfuration and tetrahydrobiopterin (BH_4_) cycles. The connections between the cycles forming the methylation pathway are shown in Figure 1.

The universal donor of the methyl group is S-adenosylmethionine (SAM), a derivative of adenosine and methionine, which is the most important substrate in methylation processes. SAM is necessary for regulating the functions of nucleic acids and many proteins that are important for proper prenatal development as well as throughout life after birth. After delivering the methyl group to target sites (e.g., DNA), S-adenosylhomocysteine (SAH) is formed, which is then transformed into homocysteine and adenosine via the enzyme adenosylhomocysteinase [6]. The SAM-to-SAH ratio has been used as a marker of “cellular methylation capacity” [7], and it has a documented role in the pathogenesis of CAD [8].

Methylation plays a role in gene expression, the functioning of multiple enzymes, the synthesis of catecholamines, and DNA repair. DNA and histone methylation occur during cell division, epigenesis, and imprinting. Maintaining the appropriate balance between substrates, products, and cofactors of the methylation pathway is essential for the homeostasis of the body. Methylation is crucial for maintaining the proper function of the vascular endothelium, the maturation and development of the central nervous system, and the regulation of gametogenesis and embryo development [9]. Moreover, recently, the folate cycle and the one-carbon cycle have been identified as regulators of the aging process [10].

Impairment of the methylation pathway leads to the development of neurological disorders such as Alzheimer’s disease, autism, Down syndrome, and multiple sclerosis; gynecological conditions such as impaired fertility and intrauterine death; as well as immune disorders such as cancer and thrombophilia; and cardiovascular diseases such as CAD and ischemic stroke [11,12,13,14,15,16,17,18,19,20,21,22].

## 3. Folate Metabolism

Folates are essential to life, serving as components and catalysts in biochemical reactions, particularly through their central role in the methylation cycle. They participate in processes crucial for the growth, development, and reproduction of all body cells, as well as in the synthesis of nucleic acids, purines, and pyrimidines. Folates also contribute to the hydroxylation of long-chain fatty acids and the proper functioning of the hematopoietic, nervous, and cardiovascular systems [26].

**Figure 2:**
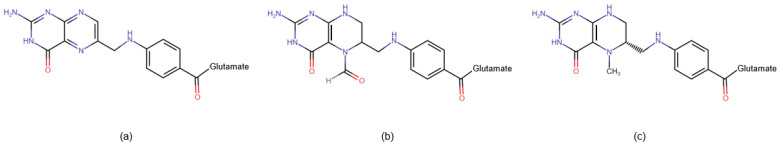
Chemical structure of folate pharmaceuticals. (**a**) Pteroylglutamic acid, the most commonly used form of folic acid, requires conversion by two enzymes: DHFR and MTHR; (**b**) Folinic acid, used in antifolates (e.g., methotrexate) overdose, requires conversion by MTHFR; (**c**) 5-MTHF, the biologically active form of folate, does not require conversion by the enzymes. Abbreviations used: DHFR—dihydrofolate reductase; MTHFR—methyltetrahydrofolate reductase.

Folates, which are chemical salts of folic acid, represent a group of pterin derivatives. Research has identified approximately 150 different forms of folate, with about 20 found in nature [27]. Humans cannot synthesize folate, and because it is water-soluble, it can be stored to a limited extent. For this reason, folate must be included in the diet or externally supplemented. Forms of folate currently available as pharmaceuticals have been presented in Figure 2. The variations among individual folate compounds lie in the oxidation level of the pyridine ring and the number of glutamic acid residues. The main folates include:

- Folic acid (also known as folacin or vitamin B9): This water-soluble molecule is predominantly found in green leafy vegetables such as asparagus, spinach, lettuce, and broccoli. In food, folic acid exists as a complex compound known as polyglutamine conjugates. These compounds are broken down in the small intestine into monoglutamates, which are absorbed into enterocytes. Within the cell, folic acid is converted to dihydrofolate (DHF) and then to tetrahydrofolate (THF) by the enzyme dihydrofolate reductase (DHFR). Subsequently, the enzyme serine hydroxymethyltransferase (SHMT) transfers the methylene group from the serine side chain to THF, resulting in the production of 5,10-methylenetetrahydrofolate (5,10-MTHF) and glycine. Methylenetetrahydrofolate reductase (MTHFR) then facilitates the formation of 5-methyltetrahydrofolate (5-MTHF), the biologically active form of folate.
- Pteroylglutamic acid (synthetic oxidized folate): This synthetically produced molecule consists of a pteroyl residue and 2 to 7 glutamine residues. It is used as a dietary supplement and for food fortification. Before entering the folate cycle, it must be reduced by DHFR to DHF and then to THF, ultimately being converted to biologically active 5-MTHF. However, synthetic folic acid is very hard for DHFR to recycle. Supplementation with synthetic folic acid may lead to a syndrome known as unmetabolized folic acid (UMFA) syndrome.
- Folinic acid (leucovorin): This synthetic molecule is a 5-formyl derivative of THF, which is converted into 5,10-MTHF without the need for DHFR. MTHFR is required for its conversion to 5-MTHF. Folinic acid is used to mitigate the toxic effects of chemotherapy agents that disrupt folate metabolism by inhibiting DHFR (e.g., methotrexate).
- 5-methyltetrahydrofolate (levomefolic acid, L-methylfolate, 5-MTHF): This represents the predominant physiological form of folate in the blood. The availability of 5-MTHF facilitates the conversion of methionine to S-adenosylmethionine (SAM), a universal methylation effector. After the release of the methyl group, S-adenosylhomocysteine (SAH) and homocysteine are produced, exerting feedback that inhibits methylation. Compared to other folates, which require conversion by enzymes, 5-MTHF is directly involved in nitric oxide production, protecting the vascular endothelium. Moreover, the novel concept of identifying CAD patients with 5-MTHF deficits may translate into the use of 5-MTHF in pharmacotherapy to restore the proper function of the vascular endothelium, but further research is needed on this topic.

### Differences between Folates—Diagnostic Insights

Limited awareness of the differences among individual compounds within the folate group and of the variations in tests used for detecting folate levels in the blood contributes to substantial misinterpretation of test results [28]. Fluorescence-based tests, commonly employed in diagnostics, evaluate levels of all folates containing a pteroyl ring. However, using such tests to monitor folate therapy does not accurately reflect the level of the biologically active form of folate. In contrast, tests utilizing liquid chromatography–mass spectrometry (LC–MS) have the capability to differentiate between 5-MTHF, pteroylglutamic acid, and folinic acid. Therefore, these tests, despite not being widely utilized yet, should be employed in assessing folate status, as they solely enable the evaluation of the concentration of the metabolically active methyl donor and provide a genuine reflection of the folate status in patients with genetic methylation disorders [29].

## 4. Pathogenesis of CAD and Its Risk Factors

CAD is usually the first and most frequent manifestation of cardiovascular disease, and it remains the leading cause of mortality and morbidity worldwide. In the European region, CAD is the most common cause of death [3]. The incidence of CAD is 2–4/1000, and the prevalence is 20–40/1000. The first cardiovascular event, e.g., myocardial infarction, can occur as early as 20 years of age, and the incidence of CAD increases with age in both sexes. According to a report published by the World Health Organization (WHO), IHD was the leading cause of death worldwide in 2000–2019, accounting for 16% of all deaths, and since 2000 there has been a global increase in the number of deaths caused by it (2 million deaths in 2000 and 9 million in 2019).

**Figure 3:**
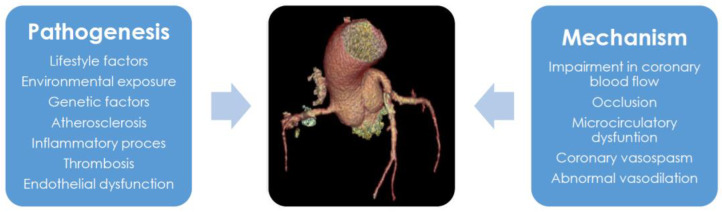
Pathogenesis and mechanisms of coronary artery disease (CAD). CAD is caused by the interplay of genetic and environmental factors leading to various underlying mechanisms, such as endothelial dysfunction and inflammatory processes. Different pathological conditions result in heterogeneous CAD mechanisms.

CAD is caused by an impairment in coronary blood flow, leading to chronic or acute ischemia of the heart muscle. Coronary blood flow impairment is typically associated with atherosclerosis, which affects the epicardial coronary arteries. However, there are many additional mechanisms leading to myocardial ischemia, such as thrombosis, coronary vasospasm, inflammation, and microcirculatory dysfunction [30] (Figure 3).

Atherosclerosis, a chronic arterial disease characterized by typical lesions due to lipid accumulation and fibrosis, is primarily driven by oxidized low-density lipoprotein particles (oxyLDL), which are involved in nearly all stages of its progression. Lipoproteins, high-molecular complexes comprising a hydrophobic lipid core containing cholesterol esters and triglycerides, along with a polar shell consisting of phospholipids, free cholesterol, and apolipoproteins, play a crucial role in lipid metabolism and transport.

While the inflammatory process within vessel walls is currently recognized as the primary cause of atherosclerosis [31], endothelial dysfunction contributes to CAD pathogenesis in its earliest stages [30]. Contrary to previous beliefs that atherosclerosis was an irreversible, age-associated degenerative process, it is now understood to develop episodically, potentially subsiding, with lifestyle modifications and medications capable of modulating its course [32].

The traditional cardiovascular risk factors were identified in the 1960s. According to the European Society of Cardiology, they are dyslipidemia, hypertension, smoking, diabetes, and obesity. The risk of CAD increases after the age of 40. These risk factors have been included in the Systemic Coronary Risk Estimation 2 (SCORE2), SCORE2-Older Persons (SCORE2-OP), and SCORE2-Diabetes cardiovascular risk classification systems [33,34]. Despite the implementation of therapy for traditional cardiovascular risk factors, such as recommendations to lose weight, stop smoking, and implement antihypertensive, lipid-lowering, and antidiabetic drugs, the therapeutic goals are not achieved by the patients from the high cardiovascular-risk group [35].

In addition to lifestyle-related risk factors, numerous genetic, biochemical, and vascular wall characteristics contribute to heightened cardiovascular risk. Physicians may consider non-traditional risk factors, including circulating biomarkers, genetic factors, psychosocial and socioeconomic factors, air pollution exposure, ethnicity, frailty, and family history. Imaging test results such as coronary artery calcium scoring, contrast computed tomography angiography (CCTA), and carotid ultrasound findings should also be factored in. Only a multidimensional approach holds promise for precise risk prediction [36].

The current approach to CAD risk factors divides them into three main categories: genetic susceptibility, environmental exposure, and lifestyle factors [37]. The individual risk of developing CAD is modulated by the interplay of genetic and lifestyle factors. The environmental factors modulate gene expression by epigenetic mechanisms [38]. Personalized risk prediction tools such as coronary artery calcium score, polygenic risk score, and metabolic risk score may improve cardiovascular risk assessment.

### 4.1. Genetics, Genomics, and Epigenetics—A Novel Approach to Understanding the Pathogenesis of CAD

CAD results from a combination of environmental and genetic factors. A comprehensive understanding of the genetic components contributing to CAD is a key objective of modern medicine, with genetic factors estimated to contribute between 40 and 60% to CAD risk [37]. The genetic underpinnings of CAD may be indicated by various factors, including early onset of CAD (before 55 years in men and 65 years in women), extensive angiographic progression, familial clustering of CAD cases, the presence of multiple risk factors in family history, and the absence of classic risk factors in affected family members [39].

The rapid growth of genetics results in complex terminology: The term genetics refers to the study of the way that certain conditions are passed down from one generation to another. Genomics describes the sequence of genetic material, maps the whole patient’s genome, and determines all relationships and interactions within the genome. Epigenetics deals with the study of changes in gene expression that do not result from sequence modifications in the deoxyribonucleotide (DNA) strand.

Understanding the etiology of CAD requires determining which genes are involved in the development or modification of the phenotype and detecting potentially harmful mutations, as well as determining the mutual interactions of individual genes and the environment.

Considering the advantages and disadvantages of these three approaches, when it comes to classical genetics, the main strength is that the polymorphisms remain unchanged throughout the patient’s life, unlike environmental risk factors such as cholesterol level, nicotine addiction, blood pressure values, and body weight. Identification of people at high risk of CAD using genetic diagnostics allows for early prevention and treatment of the disease. However, the information possessed by selected mutations and polymorphisms may be limited. On the contrary, genomics, determining all relationships and interactions within the genome, has great potential for the prediction of various diseases, but its application may be limited due to the still restricted knowledge precluding its usage in clinical practice. Epigenetics, recently gaining a lot of interest from a pathophysiological point of view, is the linkage between genetics and environmental exposure in CAD development. Epigenetics may play a key role in the development of CAD because the environment influences epigenetic patterns and can modulate gene expression [38]. Epigenetic mechanisms, such as DNA methylation, have been linked with CAD [39]. However, the DNA methylation changes are reversible and can vary over time. Epigenetics use in cardiovascular disease treatment is still in the development stage.

#### Large-Scale Research or Candidate Genes Approach?

In recent years, there has been a surge in research focusing on the genetic predisposition to CAD, particularly through large-scale studies such as genomics, transcriptomics, proteomics, and metabolomics. Genomics involves mapping the entire genome, while transcriptomics examines gene activity states, proteomics studies protein interactions, and metabolomics analyzes cellular pathway metabolites.

Genome-wide association studies (GWAS) utilize an approach that scans the entire genome for associations between single nucleotide polymorphisms (SNPs) and traits of interest. DNA microarray technology allows for the simultaneous identification of numerous SNPs, which can predict the risk of lifestyle diseases. Molecular diagnostics in this context involve determining an individual’s SNP pattern and comparing it with known patterns associated with specific diseases. SNP analysis results may potentially serve as the basis for creating genetic profiles in the future [40,41].

However, the contribution of genes identified through GWAS to CAD pathogenesis is often unclear [41]. While initially 55 CAD-related loci were identified, by 2018, the number had increased to 163 genetic variants deemed important in CAD development [42,43]. Further studies have led to the identification of over 321 CAD susceptibility loci, with ongoing efforts to identify new variants [41,42,43,44]. Despite this, these genes explain less than 20% of CAD heritability [44,45]. Notably, most genetic loci associated with CAD are not linked to traditional risk factors [45,46], and the biological functions of many gene variants remain poorly understood. However, the total risk summarized in one number called the genetic risk score (GRS) plays a role in identifying individuals at high CAD risk, allowing for early preventive strategies [44,45,46,47], and has shown promise in reducing major adverse cardiovascular events (MACE) by 40% to 50% in clinical trials [37,38].

In contrast, the candidate gene approach in genetic testing focuses on the association between genetic variation within specific genes and disease phenotypes rather than attempting to identify all SNPs associated with the disease [47,48].

The formation and progression of atherosclerotic plaques involve biochemical processes involving numerous enzymes, receptors, and their ligands, molecules encoded by various genes that interact with environmental factors. In the development of CAD, the mechanisms responsible for the inflammatory response, endothelial function, platelet function, lipid and apolipoprotein metabolism, folate metabolism, the development of thrombosis, insulin sensitivity, and mechanisms of blood pressure regulation should be taken into account [39,49].

Studies show that polymorphisms in genes encoding the folate cycle significantly contribute to atherogenesis and endothelial dysfunction [48,50]. For instance, in a meta-analysis conducted on over 87,000 participants, it was found that the T allele of the 665C>T *MTHFR* polymorphism is a risk factor for CAD, which is partially mediated by abnormal lipid levels [50,51]. Recently selected polymorphisms of the ADAMTS7 (15q24.2) gene encoding metalloproteinases (MMPs) with proteolytic activity against extracellular substrates proved to increase the risk of CAD together with total cholesterol and LDL concentration abnormalities in serum [51,52].

Selected genes responsible for the development of CAD with known pathomechanisms and taking into account the type of inheritance are presented in Table 1.

Identifying individuals at high risk of CAD through genetic diagnosis enables early preventive measures and personalized therapy, thereby improving the prognosis. As advancements in this field continue, there is potential for genetics to play a more prominent role in prevention strategies. Furthermore, the insights gleaned from individual genetic information have the potential to drive the development of new therapeutic agents [53,54].

## 5. CAD Biomarkers

Biomarkers are objectively measurable biological parameters that reflect specific physiological states of the body. They are crucial in the early detection of chronic diseases. These may result from physical tests, laboratory tests, or imaging tests [54,55].

For example, novel cardiac CT imaging biomarkers, such as the texture and density of the epicardial and perivascular adipose tissue, may be implemented in risk stratification strategies [56,57].

Recent studies show that up to 25% of patients hospitalized for first-time ACS do not have any of the four main risk factors (dyslipidemia, hypertension, cigarette smoking, diabetes) [57,58], highlighting the need to discover new biomarkers for the early identification of CAD patients and the development of new targeted therapies.

**Figure 4:**
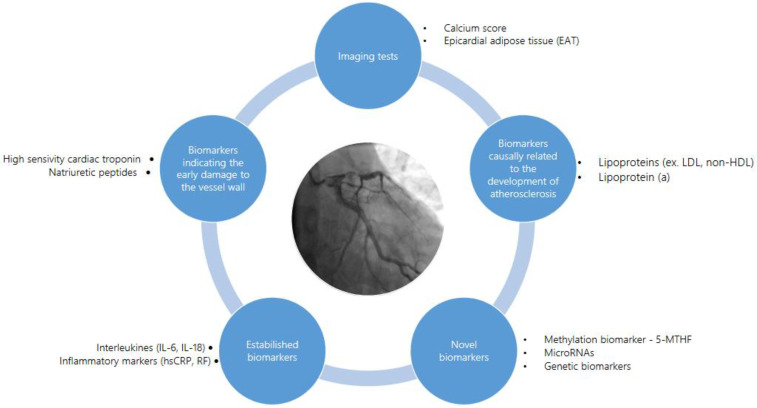
Coronary artery disease biomarkers. Selected blood and imaging biomarkers were divided by mechanism and novelty.

CAD blood biomarkers are divided into those causally related to the development of atherosclerosis and those that indicate early damage to the vessel wall [33,55] (Figure 4).

An increasingly better understanding of the pathomechanisms leading to the development of CAD and the constant improvement of research techniques result in an increasing list of new biomarkers, such as various lipoproteins and exosomes that play a role in lipid metabolism and inflammatory markers [58,59].

Specifically, in the group of patients without the classical risk factors, it is recommended to search for additional biomarkers associated with the proinflammatory state (e.g., IL-6, IL-1B, IL-18, hs-CRP, ANA, ANCA, rheumatoid factor, anti-CCP Ab), thrombotic factors (fibrinogen, PAI-1, homocysteine, haptoglobin), as well as genetics (e.g., polygenic risk scores and genetic counseling) [60,61].

Some biomarkers may have a causal relationship with the development of atherosclerosis (e.g., lipoprotein (a) reflecting the pathogenic lipid fraction) or may explain its pathogenic mechanism (e.g., C-reactive protein indicating inflammation or inflammatory hematological ratios [62,63]). Others may be markers of early heart damage (e.g., high-sensitivity cardiac troponin) or heart failure (e.g., natriuretic peptides).

Currently, many new studies involving cardiologic biomarkers concern the small noncoding parts of the RNA (MicroRNAs). The human genome responds dynamically to changes in the environment throughout a person’s life, influencing the phenotypic expression of diseases. MicroRNAs are recognized as major regulatory gene families playing a role in epigenetic regulation [49]. Due to the stable plasma concentration of miRNAs, it is possible to use them as biomarkers [59].

For example, the loss of nitric oxide synthesis, which is an initial step of atherogenesis, may be detected by the microRNA [64,65]. MicroRNAs have been proven to discriminate between unstable CAD patients and stable ones [40,65].

On the other hand, the biomarkers that have already established a position in cardiology are the proinflammatory cytokines. Cytokines are a group of protein molecules that mediate intercellular signaling between organs. They are produced by tissues and cells and are only produced in response to a stimulus. In many forms of left ventricular dysfunction, proinflammatory cytokines such as interleukin 1, interleukin 6, and tumor necrosis factor-alpha (TNF-alpha) are rapidly expressed in the myocardium, which, through specific receptors, mediate various processes such as the expression of genes, cell growth, or apoptosis [63]. In chronic ischemic cardiomyopathy and chronic LV pressure or volume overload, myocardial expression of proinflammatory cytokines is triggered by increased LV wall tension. Cytokine expression in the myocardium contributes to reduced contractility and unfavorable LV remodeling. Therefore, the increased concentration of cytokines reflects a rather late phase of myocardial damage.

### 5.1. Patomechanisms at the Early Stage of Atherosclerosis and Related Biomarkers

Currently used biomarkers are useful in identifying patients with established cardiovascular disease, but their role is partially limited due to individual differences and the lack of reflection of intracellular processes at the early stage of CAD development.

There is a lack of blood biomarkers that reflect the early stage of atherosclerosis. Modification of endothelial nitric oxide synthase (eNOS), caused by oxidative stress by uncoupling BH_4_, affects endothelial cell dysfunction and the initiation of the development of atherosclerotic plaque [57,58]. The central role of oxidative signaling in cardiovascular pathophysiology positions measurements of redox state parameters as excellent markers for research and clinical applications. For example, mitochondrial superoxide levels were recently proposed as a marker of CAD [66]. It seems crucial to identify a biomarker that correlates with the redox state and may lead to the early detection of CAD. However, despite this, no redox biomarker is currently widely used clinically, mainly due to analytical problems, including the relative instability of reactive oxygen species [61,67]. The physiologic form of folate, 5-MTHF, is involved in eNOS coupling, which results in increased nitric oxide production, protecting the vascular endothelium. However, 5-MTHF assays are currently only available as part of scientific research.

### 5.2. Public Health Perspective

According to the current ESC guidelines, in order to include a potential new marker in the assessment of cardiovascular risk, it must improve risk prediction, have an impact on public health, be feasible in everyday practice, have an impact on increasing cardiovascular risk and also on reducing cardiovascular risk, and the literature on the topic cannot be biased. Currently, only a few of the markers meet all of the required criteria at the same time.

For example, the increased concentration of lipoprotein (a), which is genetically determined and twice as common as familial hypercholesterolemia (FH), is associated with a significant risk of atherosclerotic diseases, including CAD [59,68]. Lp(a) is recognized as an independent cardiovascular risk factor, and from 2019, it is recommended to measure its concentration at least once in a lifetime in every adult and in selected people with a family history of premature cardiovascular disease [53]

Routine measurement of the non-classical biomarker concentration in blood or urine is not recommended according to the current state of knowledge [55,58]. However, in a specific group of patients, especially in patients with a history of myocardial infarction at a young age, without classic risk factors, or with a strong family history of CAD, additional tests may be justified [69,70].

This approach is used to prioritize public health interventions for primary and secondary prevention programs but does not encompass all the etiological factors of CAD.

## 6. Methylation Disorders

Methylation is influenced by various environmental (epigenetic) and genetic factors [71,72]. Externally, the proper course of methylation mainly depends on the supply of micronutrients that are donors of methyl groups (-CH_3_): choline, betaine, folacin, methionine, and the supply of cofactors such as vitamin B12, vitamin B6, vitamin C, iron, and zinc [73,74]. However, the low stability of folates results in their limited bioavailability.

The body’s ability to properly carry out methylation reactions is also influenced by abnormal functioning of the digestive system, such as intestinal malabsorption syndromes, abnormal pH in the intestine, and abnormal hepatocyte function [75,76]. Alcohol and cigarettes are among the products that negatively affect folate absorption.

The increased need for folates (e.g., during pregnancy, hemolytic anemia) and drug interactions (anti-inflammatory drugs, antiepileptic drugs, contraceptives, antimalarials, and barbiturates) are the causes of impaired methylation cycles [72,77]. Methotrexate therapy, which inhibits the conversion of dihydrofolate to tetrahydrofolate, requires folate supplementation to prevent methylation disorders [74,78].

### 6.1. Genetically Determined Methylation Disorders

The ability to methylate is genetically determined. Genetic changes in genes encoding key enzymes in the methylation process affect their activity. The proper functioning of the metabolic pathways of the folate cycle and one-carbon groups depends on identified single nucleotide polymorphisms (SNPs), which alter the activity of proteins participating in the cycle, thus impairing specific physiological functions and contributing to the pathogenesis of infertility, neurological disorders, and cardiovascular diseases [11].

For instance, thymidylate synthase (TS), which catalyzes the conversion of 5,10-MTHF to DHF and deoxyuridine monophosphate (dUMP) to deoxythymidine monophosphate (dTMP), has recently been associated with CAD susceptibility [76,79].

Methylation disorders may also arise from polymorphisms responsible for the formation of enzymatic proteins that carry out these reactions, such as the enzyme methyl-tetrahydrofolate reductase (MTHFR). Polymorphisms identified in the field of folate metabolism include:

- absorption of folate from food: enzyme encoding glutamine carboxypeptidase II—polymorphism 475H>Y (rs61886492)
- folate transport—polymorphism (rs1051266) of the RFC1 gene
- activity of folate receptors—genes encoding FOLR1, FOLR2
- folate metabolism—polymorphisms encoding the genes dihydrofolate reductase (DHFR), methylenetetrahydrofolate reductase (MTHFR), and methionine synthase (rs1805087).

#### Methylenetetrahydrofolate Reductase Gene Polymorphisms

The *MTHFR* gene is responsible for the production of 5,10-methylenetetrahydrofolate reductase (MTHFR), a key enzyme involved in folate metabolism. This enzyme catalyzes the reduction of 5,10-methylenetetrahydrofolate to the active form of folate (5-MTHF, levomefolic acid), which is fundamental in the process of homocysteine remethylation [74,78]. Single nucleotide polymorphisms (SNPs) in the *MTHFR* gene result in many variants of the enzyme, reducing its ability to generate 5-MTHF. *MTHFR* polymorphisms are associated with cardiovascular diseases, cancer, neurological diseases, diabetes, and psoriasis.

The *MTHFR* locus is located on chromosome 1 at the end of the short arm (1p36.6) [77,80]. Many variants of the *MTHFR* gene polymorphism have been described, but the most attention is paid to the c.665C>T and c.1286A>C polymorphisms. These polymorphisms can be found in heterozygous (polymorphism in only one allele), homozygous (polymorphism in both alleles), and also in the form of compound heterozygotes (one of the above-mentioned polymorphisms in one allele and the other in the second allele). Recently, the nomenclature for the most common *MTHFR* polymorphisms has changed (Table 2).

In carriers of the c.665C>T polymorphism (the terms C677T or C665T are no longer recommended; rs1801133 according to the Single Nucleotide Polymorphism Database), there is a transition from cytosine (C) to thymine (T) at nucleotide position 665 in exon 4 of the *MTHFR* gene, and consequently, the change of alanine to valine in amino acid 222 of this protein (p.Ala222Val).

In carriers of the c.1286A>C polymorphism (the term A1298C is no longer recommended; rs1801131 according to the Single Nucleotide Polymorphism Database), there is a transversion of adenine (A) to cytosine (C) at position 1286 of the *MTHFR* gene and, consequently, the replacement of glutamine with alanine at amino acid 429 of this protein (p.Glu429Ala) [78,81].

**Table 2:** Nomenclature of *MTHFR* gene polymorphisms. Based on [79,80,82,83].

	Current Nomenclature	Alleles	Past Nomenclature	Genetic “Raw Data”
	c.665C>T MTHFR polymorphism		C677T MTHFR polymorphism	
Genotypes	c.[665C=]c;[665C=]	Both “wild type” alleles	C677C	G/G
	c.[665C>T];[665C=]	One polymorphic allele: c.665 C>T heterozygote	C677T	A/G
	c.[665C>T];[665C>T]	Both polymorphic alleles: c.665 C>T homozygote	T677T	A/A
	c.1286A>C MTHFR polymorphism		A1298C MTHFR polymorphism	
Genotypes	c.[1286A=];[1286A=]	Both “wild type” alleles	A1298A	T/T
	c.[1286A>C];[1286A=]	One polymorphic allele: c.1286 A>C heterozygote	A1298C	G/T
	c.[1286A>C];[1286A>C]	Both polymorphic alleles: c.1286 A>C homozygote	C1298C	G/G

The frequency of polymorphisms varies depending on geographical location and ethnicity. They are most common in the heterozygous variant. According to the Genome Aggregation Database (gnomAD), in the general population, the polymorphic allele c.665C>T affects 30.85% of the population, while the c.1286A>C allele affects 28.58% of the population [81,84]. *MTHFR* gene polymorphisms are particularly common in Caucasians, slightly less common in Yellows, and are least common in Blacks [82,85].

Heterozygous c.665C>T occurs in 20% to 40% of the white population and 1% to 4% of most other ethnic groups. Homozygous c.665C>T occurs in approximately 10% of the general population in Europe and may affect up to 25% of individuals in some populations (Iran, China, Turkey, Spain, and southern Italy). The heterozygous variant c.1286A>C is found in 20% of Europeans, 8–15% of the general white population, and 1–4% of Asians; the homozygous variant is found in 9% of the general population [83,86].

In a multicenter study conducted in Poland in 2011, the genotype frequency of the c.665C>T polymorphism of the *MTHFR* gene was determined. For the variants found c.[665C=];[665C=], c.[665C>T];[665C=], c.[665C>T];[665C>T], in men it was 47%, 43%, and 10%, and in women 49%, 42%, and 9% [81,86]. In a 2015 study conducted on the population of Polish women, the frequency of the above c.665C>T polymorphisms was 50.60%, 39.88%, and 9.52%, respectively. However, the genotypes of the c.1286A>C c.[1286A=];[1286A=], c.[1286A>C];[1286A=], c.[1286A>C];[1286A>C] polymorphism occurred with a frequency of 42.75%, 47.88%, and 9.37% [82,85].

The combination of two variants as heterozygotes is common (>20%). Combined homozygosity for one variant and heterozygosity for another is rare but does exist: 0.4%. No double homozygosity was detected; it is probably a lethal combination [11].

The presence of *MTHFR* polymorphisms causes a loss of 40 to 70% of enzyme function in the case of the c.665C>T variant and from 30 to 50% in the case of the c.1298A>C variant [78,81]. The remaining activity of the MTHFR enzyme in the case of particular genotypes has been presented in Table 3.

A decrease in activity in terms of 5-MTHF formation leads to a decrease in the concentration of folate in the blood [84,87]. The availability of 5-MTHF plays a key role in the amount of circulating nitric oxide, and this is a mechanism independent of homocysteine concentration [70,73,75,88,89,90]. A shift in the balance between NO production and oxidative stress in endothelial cells leads to vascular endothelial dysfunction (ED), which is a key step in the initiation of CAD development.

## 7. The Role of Methylation Pathway in Endothelial Dysfunction

The endothelium is a single layer of cells that lines the inner walls of vessels. It regulates the tension of blood vessel walls and blood flow through the vessels thanks to the controlled release of vasodilators such as nitric oxide (NO), prostacyclin (PGI2), hyperpolarizing factor, and vasoconstrictors like endothelins, platelet-activating factor (PAF), thromboxane A2, and prostaglandin H2 [67,91].

The distribution of atherosclerotic lesions in the arteries is heterogeneous. Lesions appear most quickly or progress most rapidly in places of turbulent blood flow, such as divisions of the arteries. Laminar blood flow protects against the development of atherosclerosis by stimulating mechanoreceptors on the surface of endothelial cells, which results in increased expression and activation of endothelial nitric oxide synthase (eNOS).

Endothelial homeostasis is a strict balance between vasodilation and vasoconstriction, pro- and antithrombotic, pro- and anti-inflammatory, and pro- and antiproliferative processes. The endothelium has a vasoprotective function by producing and metabolizing numerous endothelial transmitters released in response to chemical and mechanical stimuli (e.g., shear forces). The actions of endothelial NO are believed to play a major role in endothelial function [68,92].

Nitric oxide is produced from L-arginine via an enzyme called nitric oxide synthase (endothelial nitric oxide synthase, eNOS). The release of NO occurs due to the constant stimulation of endothelial cells by vascular shear forces. NO activates soluble guanylate cyclase and causes an increase in cyclic guanosine monophosphate (cGMP) concentration in target cells, causing vasodilation. NO additionally reduces platelet aggregation and adhesion, prevents smooth muscle hyperplasia, inhibits leukocyte adhesion and the expression of proinflammatory cytokine genes, and prevents the oxidation of low-density lipoproteins (LDL) [70,90]. Studies have shown that patients with cardiovascular risk factors and CAD exhibit significantly lower NO concentrations and a higher concentration of endothelin compared to the control group [91,93].

A shift in the balance between NO production and oxidative stress in endothelial cells leads to endothelial dysfunction (ED) [70,90], which is considered an early phase of the development of atherosclerotic plaque [88,90,92,94]. ED is a state in which the endothelium has a limited ability to produce substances with a vasodilatory effect, resulting in an insufficient vascular response to stimuli and increasing the oxygen demand of the myocardium. Moreover, ED leads to increased penetrability, which exacerbates the penetration of proteins, including lipoproteins, into the vessel wall.

The synthesis of endothelial NO is catalyzed by eNOS, and its cofactors are tetrahydrobiopterin (BH_4_), reduced nicotinamide adenine dinucleotide phosphate (NADPH), and the active forms of vitamin B2—flavin adenine dinucleotide (FAD) and flavin mono-nucleotide (FMN). Reduced availability of NO produced from L-arginine by eNOS occurs in the case of decreased eNOS expression or activity; deficiency of the substrate—L-arginine; presence of an eNOS inhibitor in the circulation—asymmetric dimethylarginine (ADMA), which is produced as a result of the methylation of arginine; increased elimination of NO from the body—due to increased production of superoxide anion by NADPH oxidases and xanthine oxidases, producing peroxynitrite (ONOO-) and eNOS uncoupling [71,93,94].

Low levels of the active form of folate, 5-methyltetrahydrofolate (5-MTHF), have been shown to play a key role in inducing BH_4_ deficiency, which results in eNOS monomerization and the production of free oxygen radicals instead of NO (oxidative stress) [70,90]. The reduced availability of NO due to decreased levels of methylated folic acid can initiate endothelial dysfunction (ED), contributing to the development of CAD. The availability of 5-MTHF plays a key role in the amount of circulating nitric oxide, and this is a mechanism independent of homocysteine concentration [70,73,75,88,89,90]. In the endothelial cells of blood vessels, homocysteine is transformed by remethylation. The remethylation cycle of homocysteine to methionine requires the supply of methyltetrahydrofolate (5-MTHF) as a methyl donor and the presence of methionine synthase and its cofactor, vitamin B12. 5-MTHF, which is a methyl donor, cannot donate a methyl group in the case of vitamin B12 (cobalamin) deficiency. During the folate cycle, one molecule of tetrahydrobiopterin (BH_4_) is also regenerated in the form of BH_2_. BH_4_ is a cofactor for nitric oxide synthase (eNOS).

When the methylation cycle is impaired, the production of BH_4_ is significantly weakened, and as a result, the amount of nitric oxide (NO) produced is reduced. This explains the dysfunction of the vascular endothelium in metabolic disorders of the cellular methylation pathway, which is the stage initiating the development of atherosclerosis in the pathogenesis of coronary artery disease (Figure 1).

## 8. Practical Aspects of Genetics and Pharmacogenomics in CAD Patients

Despite the increased availability of genetic diagnostics, they are currently not widely used, and there are no recommendations for the routine use of genetic markers in the assessment of cardiovascular risk [33,55]. Genetic testing remains at the stage of scientific research. This is related to treatment options, as the majority of the results do not translate into clinical practice due to the lack of targeted pharmacotherapy.

Presently, the only exception in genetic diagnostics for cardiovascular diseases is in the case of monogenic inherited familial hypercholesterolemia (FH). FH is caused by mutations in the genes for the LDL receptor (LDLR—the most common cause), apolipoprotein B (apoB), and protein convertase subtilisin/kexin type 9 (PCSK9). FH may be homozygous (incidence 1:160,000–1,000,000) or heterozygous (1:200–500). Currently, genetic testing for FH is recommended only for individuals who meet any of the following criteria: serum total cholesterol (TC) ≥ 310 mg/dL (≥8 mmol/L) in an adult patient or family member; premature coronary heart disease in the patient or a member of his family (men < 55 years of age, women < 60 years of age); tendon xanthomas in you or a member of your family; sudden cardiac death of a family member at a young age [85,95]. According to current recommendations, although genetic testing may facilitate and accelerate the identification of patients, it is not required to make the diagnosis. In The Dutch Lipid Clinic Network scoring system, which contains diagnostic criteria for FH, genetic testing is not necessary [86,96].

In the case of FH, pharmacogenomics can be utilized, which matches the drug to the patient’s genotype. PCSK9 inhibitors were first included in the ESC guidelines for the treatment of lipid disorders in 2019 [52,53]. Proprotein subtilisin/kexin convertase type 9 is a protein involved in the metabolism of LDL receptors (LDLRs) by binding to them and stimulating endocytosis of the LDLR-PCSK9 complex and LDLR degradation in the lysosome. On the other hand, PCSK9 inhibitors are monoclonal antibodies to PCSK9 that lead to a reduction in LDLR degradation and cause a decrease in LDL-C concentration by an average of 60%, regardless of other lipid-lowering treatments carried out simultaneously [87,97]. PCSK9 inhibitors available in Poland are alirocumab and evolocumab, which are not reimbursed or available as part of the drug program conducted in selected metabolic disorders clinics. Due to too-narrow program criteria and the high cost of treatment when it is not reimbursed, access to effective personalized therapy remains limited.

Therapeutic decisions regarding the initiation of pharmacological treatment hinge on the overall cardiovascular risk assessment, which is informed by classical risk factors and risk-modifying factors [33]. Despite the identification of elevated serum levels of various biomarkers associated with CAD risk, regrettably, there are no specific treatments directly targeting these biomarkers to reduce CAD risk, thus limiting their clinical utility. Additionally, while gene editing preclinical studies are ongoing, the effectiveness and safety of this approach in humans require further validation [95,98].

## 9. Translating the Pathomechanism into Clinical Practice—New CAD Therapies

According to current knowledge, ED is a reversible process [96,99]. So far, all drugs inspired by genetic discoveries in CAD concern lipid metabolism. Although the clinical results are promising, they do not represent all the new pathophysiological concepts. Mechanisms leading to the uncoupling of eNOS are considered a promising therapeutic target. 3-hydroxy-3-methylglutaryl-coenzyme A reductase inhibitors (statins) commonly used in atherosclerotic cardiovascular diseases, among many beneficial effects on endothelial physiology, also include the prevention of eNOS uncoupling [97,100].

Reports suggest the use of antioxidants such as vitamins C and E, N-acetylcysteine, and glutathione, as well as BH_4_ and folic acid, to enhance vascular endothelial function [70,71,98,99,100,101]. However, direct interventions aiming to administer tetrahydrobiopterin analogs (BH_4_—eNOS cofactor) or supplementing with L-Arginine (eNOS substrate) did not yield a clearly beneficial effect [97,100].

### Folic Acid versus 5-MTHF in CAD Therapy

Pharmacological interventions in CAD typically target classic risk factors such as hypertension and dyslipidemia. According to current standards, studies on supplementation of B vitamins (B6, B12), folic acid, and vitamins C and D in patients with CAD have not demonstrated beneficial effects. However, there have been no studies conducted on the methylated forms of folic acid [33].

The recommended intake of folic acid in the adult diet is 200 μg per day, primarily from green leafy vegetables. Folic acid supplementation is well-established in gynecology and recommended before conception and during pregnancy to prevent neural tube defects (NTDs). Over 80 countries, including the USA, have implemented mandatory food enrichment of flour with synthetic folic acid for NTD prevention [101,102]. In countries without food fortification policies, such as Poland, women planning to conceive are advised to take a preventive dose of folic acid (400 μg daily) or its active metabolites.

Currently, the choice of the form of folic acid does not depend on the genotype. To prevent defects of the central nervous system in women who have previously given birth to a child with such a defect, a higher dose of folic acid of 4–5 mg should be used [82,84]. However, too-high doses of folic acid (>400 ug per day) may be potentially harmful. Supplementation with synthetic folic acid may lead to the syndrome recognized as unmetabolized folic acid syndrome (Unmetabolized folic acid Syndrome, UMFA) [103,104]. For instance, high folate concentrations were linked to gestational diabetes mellitus [102,104,105].

Clinical trials investigating the use of folic acid in the treatment of CVD have produced conflicting results, despite promising experimental data. These trials have largely failed to demonstrate the beneficial effect of folic acid supplementation on the cardiovascular system. Folic acid can enhance the expression and activation of certain genes, particularly in vascular smooth muscles, which may promote cell division and proliferation. Interestingly, there are reports indicating a significantly higher incidence of restenosis in stents among patients who used high doses (>1 mg) of folic acid following coronary artery revascularization procedures [104,106]. A meta-analysis of clinical trial data has corroborated these findings, suggesting that folic acid supplementation is not effective in preventing cardiovascular events in individuals with pre-existing vascular disease [107,108].

Given the increasing consumption of large doses (>1 mg) of folic acid through fortified foods and dietary supplements, there is a pressing need to monitor its effects closely [109,110]. The perspective on folic acid supplementation needs to shift. Instead of simply increasing the dosage, factors such as the bioavailability of folic acid and the patient’s genotype should be carefully considered initially. This approach may offer a more personalized and potentially effective strategy for managing cardiovascular health.

Folic acid remains inactive in the human body and requires conversion in the liver into its active form, 5-methyltetrahydrofolate (5-MTHF). This active molecule serves as a crucial methyl group donor (-CH_3_) in various metabolic reactions, including the biosynthesis of glycine from serine, the conversion of homocysteine to methionine, and the formation of purines and pyrimidines essential for DNA synthesis. It is fundamental to growth in the embryonic and fetal phases. In the adult human cardiovascular system, endothelial cells retain the ability to divide and move cells and require 5-MTHF for proliferation [106,111]. Because 5-MTHF does not require activation, it does not accumulate in the blood like folic acid in cases of reduced hepatic metabolism [108,112].

5-MTHF plays a key role in regulating the level of circulating nitric oxide [70,73,75,88,89,90]. It is synthesized by the enzyme methylenetetrahydrofolate reductase (MTHFR) from 5,10-methylenetetrahydrofolate (5,10-MTHF) sourced from the gastrointestinal tract. However, it is noteworthy that in about 30% of the European population, the function of this enzyme is impaired [110,113].

To determine how the *MTHFR* gene polymorphism affects methylation disorders, it seems reasonable to check its activity by measuring the 5-MTHF concentration. It has been reported that 5-MTHF levels in red blood cells represent long-term folate status and may be a more reliable marker of cellular methylation pathway disorders than homocysteine, and its deficiency is associated with CAD [111,114]. The level of the active folate metabolite 5-MTHF plays a critical role in circulating nitric oxide levels [70,90]. Low concentrations of 5-MTHF in red blood cells are found in patients with hypertension [112,115]. In a prospective cohort study conducted in 2011–2014 on 10,661 participants, an increased risk of death from any cause was found in people with low serum 5-MTHF levels (<23.9 nmol/L) [113,116].

The effect of 5-MTHF supplementation on endothelial function is independent of lowering homocysteine concentration. Folates directly interact with eNOS and improve the binding affinity of BH_4_ to eNOS, chemically stabilize BH_4,_ and enhance the regeneration of BH_4_ from BH_2_ [25]. Carriers of polymorphisms in genes related to folate metabolism or absorption may benefit from 5-MTHF instead of folic acid [114,117].

The use of 5-MTHF in several diseases has been studied in animal models. It has been shown that 5-MTHF administered intravenously in rats with acute kidney injury reduces oxidative stress by increasing glutathione concentrations and reducing renal lipid peroxidation [115,118]. Rats with developed aortic atherosclerosis treated with 5-MTHF showed a decrease in homocysteine levels, an improvement in the serum lipid profile, an increase in the expression of NO and NOS, an enhancement of the antioxidant properties of GSH activity, and a decrease in the expression of inflammatory factors (TNF-α, IL-6, and IL-1β) and endothelial cell damage factors (ET-1 and sICAM-1). 5-MTHF has been shown to have anti-inflammatory and antioxidant effects and reduce the occurrence of aortic damage [116,119].

There are single experimental studies on humans using 5-MTHF. In couples diagnosed with infertility and confirmed to have a c.665C>T polymorphism in both partners, treatment with 5-MTHF resulted in a decrease in homocysteine concentration in the blood [117,120]. 5-MTHF infusion improved impaired endothelium-dependent vasodilation in patients with familial hypercholesterolemia [118,121] and CAD [56,119]. The active circulating form of folate has been shown to increase the vascular BH_4_/BH_2_ ratio, reversing eNOS uncoupling and restoring endothelial function in coronary artery bypass grafting patients [75,89].

The most recent guidelines on food fortification now incorporate 5-MTHF into their standards [60,120]. Considering the 5-MTHFS ability to restore endothelial cell function, it seems crucial to verify its medical utility in clinical trials with CAD patients.

## 10. Conclusions and Future Directions

Currently, CAD prevention is tailored to the lifestyle but not the individual genotype of the patient. The current recommendations do not relate to the frequent methylation disorders caused by the methyltetrahydrofolate reductase gene polymorphism in CAD subjects or to the use of folates in this group of patients. Folates encompass a range of chemical compounds. However, routine clinical tests assess the total folate concentration without differentiating them or reflecting the level of the biologically active form of folate (5-MTHF). Deficiency in 5-MTHF is significant in the development of coronary heart disease, but diagnostic tests for 5-MTHF blood concentration are currently limited to scientific research. Identifying CAD patients with methylated folate deficiency may lead to the use of 5-MTHF in pharmacotherapy, but further research on this topic is necessary.

In summary, information derived from the determination of folate concentrations, including its methylated form, should not translate into supplementation or increasing the dosage of folic acid. Instead, it should focus on its bioavailability and consider the patient’s genotype in the development of personalized medicine.

Additionally, knowledge of the genetic CAD basis combined with the assessment of the phenotype based on new biomarkers may contribute to the development of pharmacogenomics, focusing on the individual selection of drugs depending on the genetic polymorphisms and mutations of a specific patient.

Future research is needed on the use of the active (methylated) form of folate (5-MTHF) in CAD patients with methylation disorders caused by *MTHFR* gene polymorphisms.

## Figures and Tables

**Table 1:** Selected genes and their polymorphic variants participate in the development of coronary artery disease [39,44,45,49,52,53].

Type of Inheritance	Genes, Enzymes, Receptors, and Ligands	Clinical Implications
Monogenic inheritance. Among monogenic mutations, most are involved in lipid metabolism.	Genetic causes of elevated LDL cholesterol	
	LDLR, APOB, PCSK9	Familial hyperlipidemia
	USF1	Familial combined hyperlipidemia
	Genetic causes of reduced HDL cholesterol levels	
	APOA1	Primary hypoalphalipoproteinemia
	ABCA1	Tangier disease
	LCAT	Norum disease, Fish eye disease
	ABCG5/8	Sitosterolemia
	Genetic causes of hypertriglyceridemia	
	LPL, APOC2, APOAV, GPIHBP1, LMF1	Familial chylomicronemia syndrome
	APOA1/C3/A4/A5	Familial combined hyperlipidemia and familial hypertriglyceridemia
	ATHS	Atherogenic lipoprotein phenotype
Polygenic inheritance. Mutations and polymorphisms of genes encoding enzymes, receptors, and ligands play a role in the development of atherosclerosis.	APOE, APOB, LPL, OLR1 (LOX1), SORT1, TRIB1	Lipid and apolipoprotein metabolism
	E-selectin, P-selectin, Interleukin 6, Paraoxonase data	Inflammatory response
	Connexin 37, eNOS, metalloproteinase 9, stromelysin 1	Endothelium function
	GP Ia/II receptor, GP IIIa receptor	Platelet function
	Factor V Leiden, prothrombin 20210A, protein C deficiency, protein S non-deficiency, anti-thrombin deficiency	Thrombosis and fibrinolysis
	MTHFR	Folate metabolism
	ACE, AGTR1	Blood pressure regulation

ABCA—ATP-binding cassette transporter; ABCG5/8—ATP-binding cassette sub-family G member 5/8; ACE—angiotensin-converting enzyme; AGTR1—angiotensin-II receptor 1; APOA1—apolipoproteine AI, APOA1/C3/A4/A5 gene cluster; APOA5—apolipoproteine A-V; APOB—apolipoproteine B; APOC2—apolipoproteine C-II; APOE—apolipoproteine E; ATHS—atherosclerosis susceptibility; eNOS—endothelial nitric oxide synthase; GPIHBP1—Glycosylphosphatidylinositol Anchored High Density Lipoprotein Binding Protein 1; LCAT—Lecithin–cholesterol acyltransferase; LDLR—low-density lipoprotein receptor; LMF1—Lipase Maturation Factor 1; LPL—lipoprothein lipase; MTHFR—Methylenetetrahydrofolate reductase; OLR1 (LOX1)—Oxidized low-density lipoprotein receptor 1; PCSK9—proprotein subtylisine/keksine convertase; SORT1—Sortilin 1; TRIB1—Tribbles Pseudokinase 1; USF1—upstream stimulatory factor 1.

**Table 3:** Activity (%) of methyltetrahydrofolate reductase (*MTHFR*), depending on the presence of c.665C>T and c.1286A>C polymorphisms [82,85].

Genotype		*MTHFR* c. 665 C>T		
		c.[665C=];[665C=]	c.[665C>T];[665C=]	c.[665C>T];[665C>T]
*MTHFR* c. 1286A>C	c.[1286A=];[1286A=]	100	60–70	30–40
	c.[1286A>C];[1286A=]	70–80	50–60	-
	c.[1286A>C];[1286A>C]	50–60	-	-
